# Primary Aneurysmal Bone Cyst of Sacrum: A Case Report of a 12 years old boy from Pakistan

**DOI:** 10.12669/pjms.40.12(PINS).10988

**Published:** 2024-12

**Authors:** Haseeb Mehmood Qadri, Arham Amir Khawaja, Raahim Bashir, Asif Bashir

**Affiliations:** 1 Dr. Haseeb Mehmood Qadri Post Graduate Resident, Department of Neurosurgery, Unit-I, Punjab Institute of Neurosciences, Lahore, Pakistan; 2 Dr. Arham Amir Khawaja Department of General Surgery, Shaikh Zayed Hospital, Lahore, Pakistan; 3 Dr. Raahim Bashir Research Intern, Department of Neurosurgery, Unit-I, Punjab Institute of Neurosciences, Lahore, Pakistan; 4 Prof. Dr. Asif Bashir, Department of Neurosurgery, Unit-I, Punjab Institute of Neurosciences, Lahore, Pakistan

**Keywords:** Aneurysmal bone cyst, Children, Embolization, Giant cell tumour

## Abstract

Aneurysmal bone cysts are locally invasive, benign lesions usually found in the spine or metaphysis of long bones. They can be primary (idiopathic) or secondary to other bone pathologies. Primary aneurysmal bone cyst usually occurs in the first two decades of life.

We report a 12 years old male, a known case of Type-1 diabetes mellitus, with lower back pain radiating to the right lower limb for the past two months following a fall. There were no neurological deficits, although straight leg raises and Faber’s tests were positive. Neuroimaging suggested an enhancing 7 x 8 mm lesion involving the lamina of the first sacral vertebra with protrusion into the spinal canal. Bone scan ruled out metastatic pathology. Excision of the lesion with laminectomy and foraminotomy was done. Histopathology was suggestive of aneurysmal bone cyst. The patient is living a healthy life. Upfront surgical excision without preoperative embolization and expensive medical therapies can be an option in resource-limited settings.

## INTRODUCTION

Aneurysmal bone cysts (ABC) are a common type of benign bone tumours usually located in the spine or metaphysis of long bones. They are vascular lesions that can either be primary (idiopathic) or secondary to other bone pathologies. Despite their benign nature, ABCs can be locally invasive and have a high recurrence rate.^1^ Although they make up 15% of all primary spinal bone tumours, the sacrum is a rare location (3%) for ABC and is associated with higher morbidity.^2^

The imaging modality of choice for sacral ABC is a spinal MRI. This will show the septated cystic lesion with fluid-fluid levels representing blood.^3^ Management involves surgical curettage with or without adjuvants such as bone cement, argon beam, ethanol, and cryotherapy^4^. Other less invasive treatments include aggressive biopsy, arterial embolization, sclerotherapy, and RANKL inhibitors such as Denosumab.^4^

We aim to document this case-report to bring awareness regarding the management of this unusual pathology in resource-limited settings. Scientific literature is absent on this topic in Pakistan. A Pakistani study documented in 2021 on primary ABC of the lumbar spine remains the only such published case.^5^ Nevertheless, a comprehensive search of PubMed Central, Google Scholar and SCOPUS revealed no studies on sacral aneurysmal bone cysts in Pakistan.

## CASE PRESENTATION

A 12 years old boy, a known case of Type-1 diabetes mellitus, presented to a private sector hospital in November 2023 with complaints of pain in the lower back radiating to the right lower limb for the past two months. He had a history of ground fall while running about two and a half months back. He sustained minor abrasions to the right flank for which he was treated conservatively then. The pain was dull, continuous and marked at night and with sitting posture. The central nervous system functions were normal upon complete examination and there was no apparent lesion in the lower back or right flank. His gait was normal. The straight leg raise test was positive at 45°, while the right-sided Faber’s test was positive for pain upon stimulus. Initial suspicions included soft tissue injury, non-displaced sacral or acetabular fracture, and sacroiliitis.

The patient was subjected to a battery of investigations. Radiograph lumbosacral (LS) spine was normal. Contrast-enhanced computerized tomography (CE-CT) LS spine showed a 7 mm x 8 mm, ill-defined, eccentrically placed, lytic lesion involving the right lamina of the first sacral vertebra with protrusion into the spinal canal (Fig.1A). Contrast-enhanced magnetic resonance imaging (CE-MRI) of the LS spine showed findings consistent with CE-CT; an enhancing lesion with surrounding marrow oedema, right paraspinal soft tissue enhancement, and displacement of the right nerve roots anteriorly (Fig.1B). Metastatic bone disease and locally invasive pathology, like giant cell tumour, osteoid osteoma and aneurysmal bone cyst were the differentials under consideration.

**Fig.1A:**
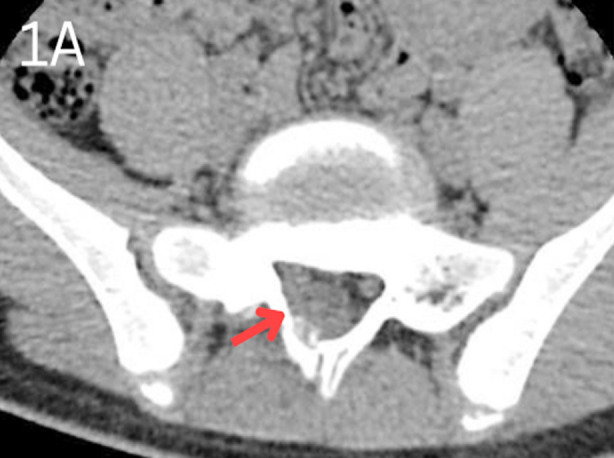
Contrast-enhanced computerized tomography (CE-CT) scan of sacrum and sacro-iliac joints. Red arrow indicates a well-defined lytic lesion of S1 lamina.

**Fig.1B:**
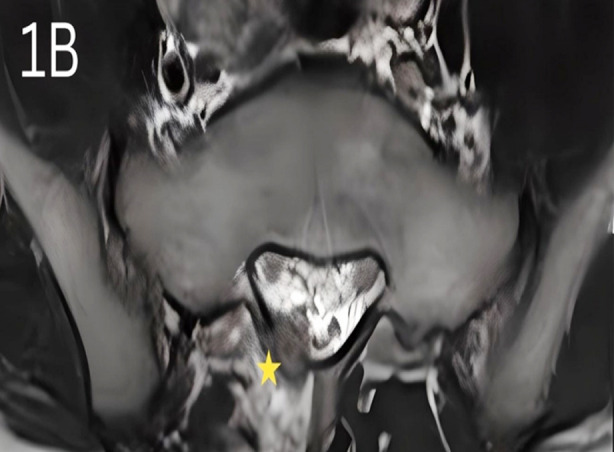
Contrast-enhanced MRI of Lumbosacral spine showing an enhancing lesion marked by a yellow star.

A Tc-99m methyl diphosphonate (MDP) bone scan revealed intense focal radiotracer uptake in the lesion. However, there was essentially normal and bilaterally symmetrical radiotracer distribution in the rest of the axial and appendicular skeleton. The whole-body bone scan ruled out the possibility of metastatic bone disease. The differentials were narrowed to locally invasive pathologies.

**Fig.2:**
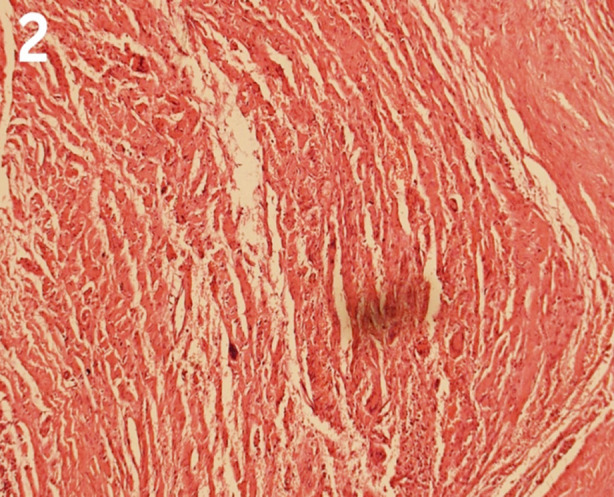
Histopathology reveals fibrous septa containing fibroblasts and giant cells.

Surgical intervention under general anaesthesia was planned. The patient underwent laminectomy and right-sided foraminotomy at L5/S1 vertebral levels. The lesion was excised completely with minimal bleeding from the site. There were no significant intra-operative complications and his recovery from general anaesthesia was smooth. However, he had poor glycemic control for which an endocrinologist and a dietician were taken on board to plan his dietary intake. He was discharged the following day with no immediate post-operative complications. Meanwhile, the histopathology report finalized the diagnosis of aneurysmal bone cyst (Fig.2). The patient followed up at our institution in February 2024 due to “affordability” issues with the complaint of pain at the incision site after two weeks. We found that the primary wound healing was delayed due to inadequate glycemic control. There was no surgical site infection. His parents were reassured and asked to follow up with post-operative neuroimaging, but they declined to get the CE-CT done. At three months follow-up, the patient has no neurological deficits and is living a healthy life with adequate dietary measures. We conclude it to be a case of primary aneurysmal bone cyst of the sacrum.

### Consent and Ethical Permission:

Patient confidentiality is maintained and written consent for the publication of patient details and clinical pictures in this journal has been obtained from the parents of the patient and can be given as required.

## DISCUSSION

It is important to differentiate between primary aneurysmal bone cysts (pABC) and secondary aneurysmal bone cysts (sABC) to devise an objective-oriented management plan for a patient. In general, pABC occurs in the first two decades of life, while the secondary ones occur after 30 years of age. Metaphysis and epiphysis are usually involved by pABC. This variability in the site of occurrence of sABC is compatible with the fact that sABC is secondary to giant cell tumour, fibrous dysplasia and chondroblastoma.^1^

The patient in the current study is a 12 years old boy with type-1 diabetes mellitus since birth. This age is in agreement with the findings in a recent Chinese study by Wang et al. where the average age of 11 adolescents with pABC was 13 years.^2^ Generally, there is no gender predilection of ABCs in human beings.^3^ However, Deventer et al. document the presence of sacral pABC in 65% of females.^4^ This implies a preponderance of sacral pABC in the female population.

Lemos et al. described pABC with heterogenous, lytic presentation and fluid levels.^5^ Our patient’s pathology presented as a lytic lesion on CT and an enhancing lesion with soft tissue involvement on MRI, which is inconsistent with radiological findings of pABC in the literature, as pABC are thought to restrict themselves within cortical bone.^1^ The paradoxical presentation on radiology in our patient signifies the atypical involvement of bone as well as soft tissue even in primary cases.

Wide excision with surrounding normal bone is a method commonly employed for ABC which is curative but debilitating.^6^ In 2009, Brastianos et al. performed either resection or curettage in their seven patients diagnosed as primary aneurysmal bone cyst and most of them had a disease-free follow-up ranging from two to five years. 7 However, two other patients underwent embolization for sacral ABC at some point in the course of their treatment and had persistent bladder and bowel dysfunction.^7^ With the rise of minimally invasive approaches in surgery, conservative management and medical treatment of bony lesions is also proving effective. Selective arterial embolization (SAE) and aortic balloon occlusion (ABO) are indicated in ABCs with high vascularity and bigger sizes. We did not opt for these investigations owing to a small size of 7 mm x 8 mm.

A group of Chinese interventionists embolized nine patients with sacral pABC using polyvinyl alcohol over a minimum of three sessions; about 44% of their patients acquired complete calcification of tumour at two years follow-up.^8^ A recently published German study describes the off-label benefits of Denosumab, a human monoclonal antibody, in treating ABCs in vital locations. pABCs of the pelvis and long bones healed well at a two-year follow-up, but recurrence and severe rebound hypercalcemia were also seen.^9^ Considering the number of sessions required for embolization and the cost of Denosumab, low-middle-income countries cannot offer these modes of treatments to presenting patients due to the limitations of resources.

In an interesting case series of 10 patients, SAE and ABO were used preoperatively and intralesional curettage was done yielding no local recurrence at an average follow-up duration of 6.5 years. Wang et al. advocate surgery for sacral pABC.^2^ Laminectomy of the first sacral vertebra and foraminotomy was offered to our patient and he is being followed every month with a good recovery. However, long-term follow-up of the patient is yet to determine the possibility of recurrence in distant future.

## CONCLUSION

Primary aneurysmal bone cyst is a benign, locally aggressive, vascular lesion that tends to cause symptoms out of proportion to the size of the lesion and mimics malignancy. Whole body bone scan is the single most important investigation for screening osteolytic and osteoblastic bone lesions for loco-regional or distant bony lesions. Our case report justifies that upfront excision of the lesion offers the advantages of minimum risk of recurrence, lesser expense, less exposure to ionizing radiation, fewer hospital visits, fewer adverse effects, and no compliance problems associated with medical therapy. For sub-centimetric lesions, it is imperative that an excisional biopsy is carried out upfront rather than resorting to serial radiological investigations to ensure quick alleviation of symptoms and bear low costs.

### Authors Contribution:

**HMQ** conceived and designed the study, did literature search, prepared the draft and critically reviewed the manuscript.

**AAK** contributed to literature review, data collection, manuscript writing and is responsible for the integrity of the study.

**RB** contributed to literature review and manuscript writing.

**AB** supervised the project and critically reviewed the article.

All authors have approved the final version of the manuscript.
